# Measurement bias in activation-recovery intervals from unipolar electrograms

**DOI:** 10.1152/ajpheart.00478.2014

**Published:** 2014-11-14

**Authors:** David Western, Ben Hanson, Peter Taggart

**Affiliations:** ^1^Department of Mechanical Engineering, University College London, London, United Kingdom;; ^2^Department of Mechanical Engineering, University of Bristol, Bristol, United Kingdom; and; ^3^Neurocardiology Unit, University College London Hospitals, London, United Kingdom

**Keywords:** unipolar electrogram, action potential duration, activation-recovery interval, repolarization, bidomain modeling

## Abstract

The activation-recovery interval (ARI) calculated from unipolar electrograms is regularly used as a convenient surrogate measure of local cardiac action potential durations (APD). This method enables important research bridging between computational studies and in vitro and in vivo human studies. The Wyatt method is well established as a theoretically sound method for calculating ARIs; however, some studies have observed that it is prone to a bias error in measurement when applied to positive T waves. This article demonstrates that recent theoretical and computational studies supporting the use of the Wyatt method are likely to have underestimated the extent of this bias in many practical experimental recording scenarios. This work addresses these situations and explains the measurement bias by adapting existing theoretical expressions of the electrogram to represent practical experimental recording configurations. A new analytic expression for the electrogram's local component is derived, which identifies the source of measurement bias for positive T waves. A computer implementation of the new analytic model confirms our hypothesis that the bias is systematically dependent on the electrode configuration. These results provide an aid to electrogram interpretation in general, and this work's outcomes are used to make recommendations on how to minimize measurement error.

the following paragraphs provide the background and objectives for this study.

The unipolar electrogram (UEG) is well established as a useful tool for studying cardiac electrophysiology (4, 10, 11, 13, 15). The morphology of UEGs is regularly used to guide catheter ablation procedures. The signals can also be used to calculate proxy measures of local depolarization and repolarization time, thus offering a convenient means of performing in vivo mapping of the spatiotemporal distribution of electrophysiological activity in human and animal hearts. The UEG is thus an important link between in silico modeling and in vitro animal experimentation and live human models.

Numerous theoretical expressions have been presented to explain how UEG morphology relates to the spatiotemporal distribution of activity in the myocardium (2, 8, 14, 17, 19). However, the interpretation of experimental recordings is not always adequately informed by the available theory, as highlighted in a recent debate regarding the estimation of repolarization times from electrograms with positive T waves (4, 5, 25). A plausible reason for the underapplication of theory to experimental recordings is that the existing expressions cannot succinctly explain how the experimental measurement configuration (such as electrode size and positioning relative to the surrounding tissue structure) influences the observed signal.

In this article, we develop a new analytic expression for the UEG that is well suited to the assessment of such influences. Based on examination of this new expression, we identify a mechanism by which measurement bias can arise in activation-recovery intervals calculated from positive (upright) T waves; we hypothesize that the bias is systematically dependent on the electrode configuration used.

To investigate this hypothesis, the new theoretical expression is implemented in a computer-simulated experiment that evaluates a range of electrode configurations. The experiment confirms the usefulness of the newly developed analytic formulation of the UEG, which can provide an improved understanding of the relationship between local tissue activity and the measured UEG and can be interpreted for recommendations of best practice in experimental work.

The following paragraphs provide methods for defining activation-recovery intervals from UEGs.

The term “activation-recovery interval” (ARI) was first used by Millar et al. (13). The ARI is well established as an important surrogate measure of the action potential duration (APD) in a region of myocardium (1, 4, 10, 13, 23). It is calculated as the interval between a nominal local depolarization time and repolarization time, which are identified based on key electrogram features.

The timing of local depolarization can be identified in the UEG by the steepest downward slope in the activation complex, d*V*/d*t*_min_. This approach is widely accepted (1, 13, 20, 21). However, two different conventions have been widely used as indexes of repolarization time: the Wyatt method (23) and the “alternative” method (1).

The Wyatt method identifies the local repolarization time as *T*_up_, the point in the T wave with the maximum upward slope. This selection is based on the reasoning that this upward slope is imposed by phase 3 of the local action potential (as manifested in the local component *UEG*_L_ or *UEG*_LS_). *T*_up_ has shown strong correlation with *t*_R_, the local repolarization time defined as the point of maximum downward slope in the transmembrane action potential (10). Also, APDs calculated using *T*_up_ are strongly correlated with the effective refractory period of the tissue (13).

Chen et al. (1) noticed a discrepancy between ARIs measured by the Wyatt method and MAP_90_. MAP_90_ is a surrogate measure of APD_90_, the time at which the action potential reaches _90_% repolarization from plateau potential to resting potential, as shown in Fig. 1. In this and subsequent studies (6, 24), it was suggested that, to achieve good agreement between ARI and APD_90_, the ARI should be measured differently for positive T waves compared with negative and biphasic T waves; the authors advocated the use of *T*_down_, the point of most negative downslope (Fig. 1), in place of *T*_up_ for calculations involving positive T waves.

**Fig. 1.**
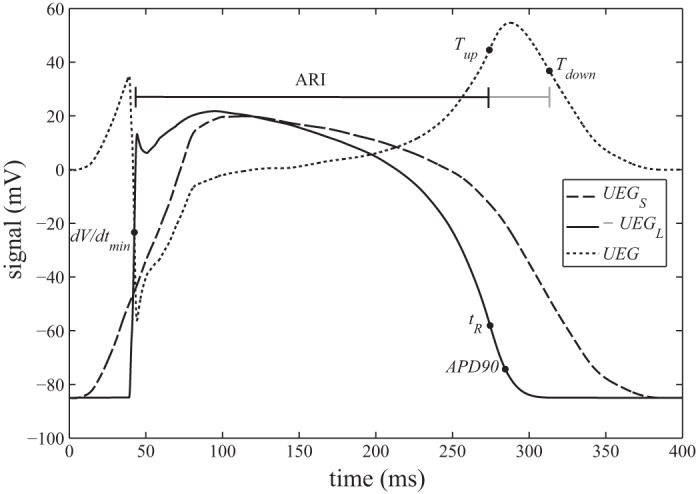
The solid trace shows a ventricular action potential calculated from the Ten-Tusscher-Noble-Noble-Panfilov model. The dashed trace shows a typical morphology of the UEG's surface (remote) component. Both are digitized with permission from Fig. 6 in Ref. 19. The dashed trace shows the UEG calculated as the difference between the other 2 traces (i.e., using the 2-component model proposed in Ref. 19). Black dots mark the commonly used indexes of activation and recovery time or depolarization and repolarization. The ARI is typically calculated as the interval between d*V*/d*t*_min_ and *T*_up_. Some researchers have used *T*_down_ in place of *T*_up_.

Although this alternative method for measuring ARIs gives, in some studies, a better approximation of when APD_90_ occurs, it lacks the theoretical foundation of the Wyatt method, which in any case is designed to indicate *t*_R_, not MAP_90_, with the assumption that *t*_R_ occurs near APD_50_. Furthermore, it has been shown experimentally that the downstroke of a positive T wave does not reliably coincide with the membrane currents associated with local repolarization (4). Computer modeling studies (19, 20) have also concluded that the alternative method does not directly reflect local activity.

In spite of the sound reasoning and experimental evidence supporting the use of the Wyatt method, the fact remains that some studies (1, 24) have observed a substantial measurement bias in the sense that, at sites repolarizing relatively early (which generally yield positive T waves), *T*_up_ tends to underestimate *t*_R_. A less substantial manifestation of this bias is clearly observable in the computational results of Colli Franzone et al. (Fig. 4 in Ref. 3) and Scacchi et al. (Figs. 3 and 8 in Ref. 20). For the Wyatt method to be applied with confidence, the mechanism by which this bias arises must be understood. In this article, a new theoretical expression of the UEG is developed and used to explain the origin of positive T-wave bias. The new expression is implemented in a computational experiment to investigate the predicted behavior.

## MATERIALS AND METHODS

### Theoretical Modeling

**Fig. 2.**
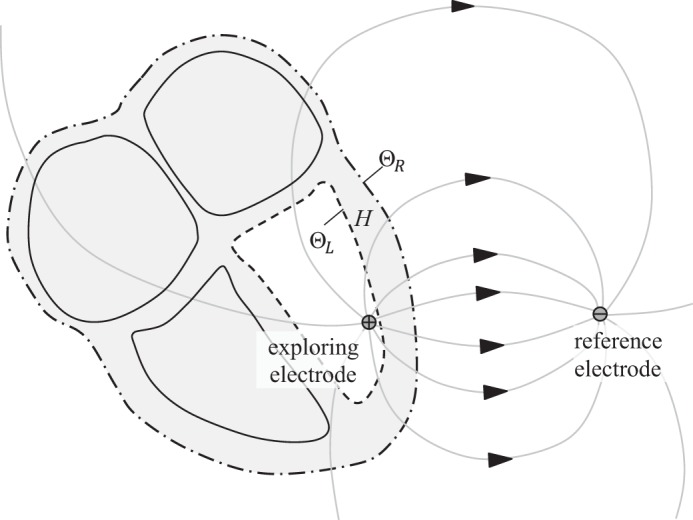
Conceptual diagram of the lead field for an intracardiac electrogram. The lead field lines pass from the exploring electrode (positive terminal) to the reference electrode (negative terminal). For in vivo recordings, the reference electrode may be positioned inside the body (e.g., a catheter electrode in the vena cavae) or on the skin. By incorporating an interior boundary surface Θ_L_ into the definition of the volume *H*, the exploring electrode is excluded from *H*. This approach allows the local component *UEG*_L_ to be replaced by the local surface component *UEG*_LS_ (*Eq. 3*) in the analytic model of the electrogram. Note that Θ_R_ and Θ_L_ are both nonintersecting (and, in this case, closed) surfaces.

According to the derivation in the appendix, the UEG can be expressed in terms of two components: an integral over the heart's outer surface, Θ_R_, and another over the surface of the endocardial chamber that contains the exploring electrode, Θ_L_ (see Fig. 2; for mid-myocardial and epicardial electrode positions, see appendix).

(1) $$UEG=UEG_{\text{RS}}+UEG_{\text{LS}}$$

(2) $$UEG_{\text{RS}}=\int_{\Theta_{\text{R}}}\alpha V_{\text{m}}M\nabla Z\cdot \text{d}\overset{⃑}{S}$$

(3) $$UEG_{\text{LS}}=\int_{\Theta_{\text{L}}}\alpha V_{\text{m}}M\nabla Z\cdot \text{d}\overset{⃑}{S}$$

*UEG*_RS_ and *UEG*_LS_ are referred to as the “remote surface component” and the “local surface component,” respectively. $\text{d}\overset{⃑}{S}$ is the outward vector perpendicular to each infinitesimal surface element of Θ. α relates the conductivities of the intracellular (i) and extracellular (e) domains in the direction transverse (*t*) to the fiber orientation: α = σ_t,i_/(σ_t,i_ + σ_t,e_). *V*_m_ is the membrane potential of myocytes at a given point in the myocardium. *M* is the conductivity tensor, describing the anisotropic conductivities at each point (2).

∇Z is known as the lead field for the electrode configuration. This lead field describes the sensitivity of the electrogram to activity at each point in the myocardium. To some extent, the nature of the lead field can be intuitively predicted; according to the principal of reciprocity (12), ∇Z is simply the negative of the electric field that would be induced by passing a unit current between the UEG electrodes (7). It is intuitive that the current flowing through the torso in this scenario would be stronger in the more conductive pathways (e.g., intracavitary blood) and would tend to circumvent regions of low conductivity (e.g., lungs). The electric field would behave similarly (except in regions of extremely anisotropic conductivity). A qualitative prediction of the lead field's behavior can therefore be achieved by envisioning the field lines or current distribution that would result from a current applied between the two electrodes.

When the exploring electrode is close to the endocardial wall, Θ_L_, the lead field ∇Z will be much stronger along the closest portion of that surface, such that activity in this region dominates *UEG*_LS_. In that sense, *UEG*_LS_ captures the influence of local activity on the UEG. Hence, it is referred to as the local surface component. In previously derived expressions (see appendix), the UEG's local component was expressed in terms of the action potential morphology at an infinitesimal point in the myocardium. For analytic purposes, *UEG*_LS_ offers several advantages over such formulations. First, it reflects the fact that the UEG receives a weighted sum of contributions from across the local tissue region. It also allows assessments of the influence of the electrode configuration, tissue properties, and tissue structure.

It can be inferred that *UEG*_LS_ ordinarily has the morphology of an inverted action potential; assuming the exploring electrode is the anode, the lead field ∇Z and elemental surface vector $\text{d}\overset{⃑}{S}$ will typically be oriented in approximately opposite directions in the dominant region of Θ_L_, so that their dot product $\nabla Z\cdot \text{d}\overset{⃑}{S}$ is negative.

The remote surface component, *UEG*_RS_, captures remote influences on the UEG. In healthy cases, it can be expected to have the morphology of a smoothed action potential, as demonstrated by Potse et al. (19) and explained in the appendix. This observation is extremely useful in enhancing the practicality of component-based analytic interpretations of UEG morphology, at least in healthy cases.

### Implementation of the New Analytic Model to Identify the Mechanism for the Positive T-Wave Bias

As noted previously, Potse et al. (19) demonstrate that positive T waves occur when local tissue repolarizes relatively early, compared with the bulk of the myocardium. While the upward slope of this T wave is predominantly a manifestation of the electrogram's local component, Fig. 1 shows that it coincides with a period of downward curvature (decreasing slope) in the remote component. The effect of this varying-slope contribution is that *T*_up_ in the electrogram occurs earlier than *t*_R_ in the local component. In negative T waves, this bias might occur in the opposite sense if *t*_R_ coincides with the later upward-curving portion of the remote component.

Potse et al. note that, in their computational study, the difference between *T*_up_ and *t*_R_ induced by this effect was “generally less than 2 ms.” They also point out that larger differences can be expected near failing, ischemic, or atrial myocytes, for which repolarization is more gradual. In practice, however, the distorting effect of the remote component could be more substantial even when recording from healthy ventricular myocytes with sharply defined repolarization phases. This point can be clarified by considering the alternative expression proposed for the electrogram's local component in *Eq. 3* (*UEG*_LS_). In this expression, the local component is not a single action potential, but a weighted average across a distribution of imperfectly synchronized action potentials. This component can be assumed to have the appearance of a smoothed action potential. Increasing the distance between the exploring electrode and the tissue surface would yield a less focused distribution of lead-field lines (the weightings of those contributions) at the nearby surface, giving *UEG*_LS_ a more smoothed appearance with a less steep repolarization upstroke. The reduced sharpness would make *T*_up_ more susceptible to the distorting effect of the remote component. One could then expect errors in estimating repolarization time to be much greater than the 2 ms suggested by Potse et al. when treating the local component as a sharply defined single action potential.

### Computer Implementation of the Analytic Model

A simple computational experiment was used to test the hypothesis developed in the preceding section: that positive T-wave bias arises when the lead field is not adequately focused on the underlying myocardium, such that the electrogram receives similarly weighted contributions from a broader region of tissue, across which the timing of repolarization is more dispersed.

Electrograms were calculated using an implementation of the two-component model expressed in *Eq. 1*. The remote component's morphology remained as shown in Fig. 1 (dashed trace, digitized from Ref. 19) to ensure that this component accurately resembled that of a whole heart model. Rather than using a single action potential for the local component's morphology, we calculated the local surface component, *UEG*_LS_, using the simplest possible model that could incorporate local dispersion of repolarization time. The action potential morphology depicted in Fig. 1 (solid trace) was assumed to propagate across a 40 mm × 40 mm square region of myocardial surface in the *x* direction, and *Eq. 3* was used to calculate *UEG*_LS_. Thus the tissue is implicitly assumed to be homogeneous and electrotonic effects are ignored. Two different values were used for the “propagation velocity” attributed to this activity, which determines the level of local dispersion of repolarization. For the high repolarization dispersion case, a propagation velocity of 65 cm/s was used, reflecting a typical activation propagation speed. Ventricular repolarization is typically found to be more synchronous than activation. Hence, a low repolarization dispersion case was simulated by using a propagation velocity of 130 cm/s.

**Fig. 3.**
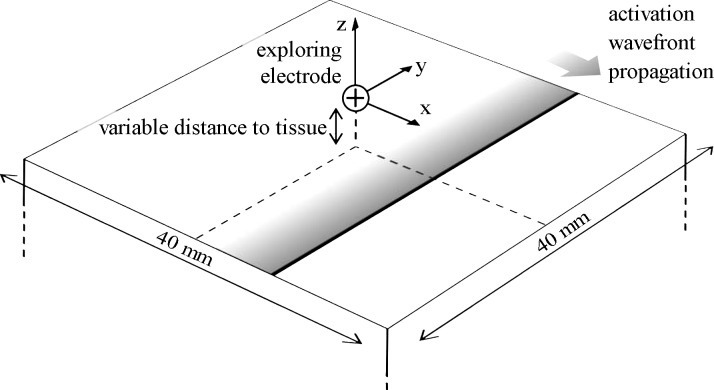
An illustration of the computational model. Note that because a typical morphology is assumed for *UEG*_RS_ rather than calculating it directly, the slab thickness does not need to be defined.

The exploring electrode was assumed to be positioned at the origin [(*x*,*y*,*z*) = (0,0,0)], directly over the center of the surface (see Fig. 3). The distance *z* to the surface varied from 0.004 to 16 mm. For simplicity and generality, the lead field ∇Z was taken as that which would occur in an infinite, homogeneous, isotropic conductive medium (18):

(4) $$\nabla Z=\frac{-\hat{r}}{4\pi \sigma \left(x^{2}+y^{2}+z^{2}\right)}$$

Here, $\hat{r}$ is the unit vector from (*x*,*y*,*z*) to the exploring electrode at the origin. σ Is the homogeneous and isotropic conductivity of the medium.

For further simplicity, the tissue was assumed isotropic as well as homogeneous, such that *M* can be replaced with a scalar factor of 1. *Equation 3* then becomes

(5) UEGLS=−α4πσ∫ΘLVmx2+y2+z2r^⋅dS⃑

Here, the variables α and σ can be assigned constant values representative of typical physiology. Because a generic morphology has been adopted for *UEG*_RS_, these values should be chosen such that the magnitude of *UEG*_LS_ realistically matches that of *UEG*_RS_. It can be assumed that there is no net transfer of charge between the electrodes during a full cardiac cycle length (duration = CL). The time integral of the electrode current through this period is therefore equal to zero. Therefore, neglecting capacitive effects (i.e., assuming voltage to be proportional to current), the time integral of the electrode voltage can also be treated as zero.

(6) $$0=\int_{0}^{\text{CL}}UEG\;\text{d}t={\int_{0}^{\text{CL}}UEG}_{\text{RS}}\text{d}t+{\int_{0}^{\text{CL}}UEG}_{\text{LS}}\text{d}t$$

By substituting *Eqs. 5* into *6* and rearranging, the factor σ/α can be separated.

(7) σα=−σα∫0CLUEGLSdt∫0CLUEGRSdt=14π∫0CL∫ΘLVmx2+y2+z2r^⋅dS⃑ dt∫0CLUEGRSdt

The right-hand side of *Eq. 7* can be calculated (in discrete form) from the model described in the previous paragraphs, to solve for σ/α. This value can then be used in *Eq. 5* to calculate *UEG*_LS_.

## RESULTS

**Fig. 4.**
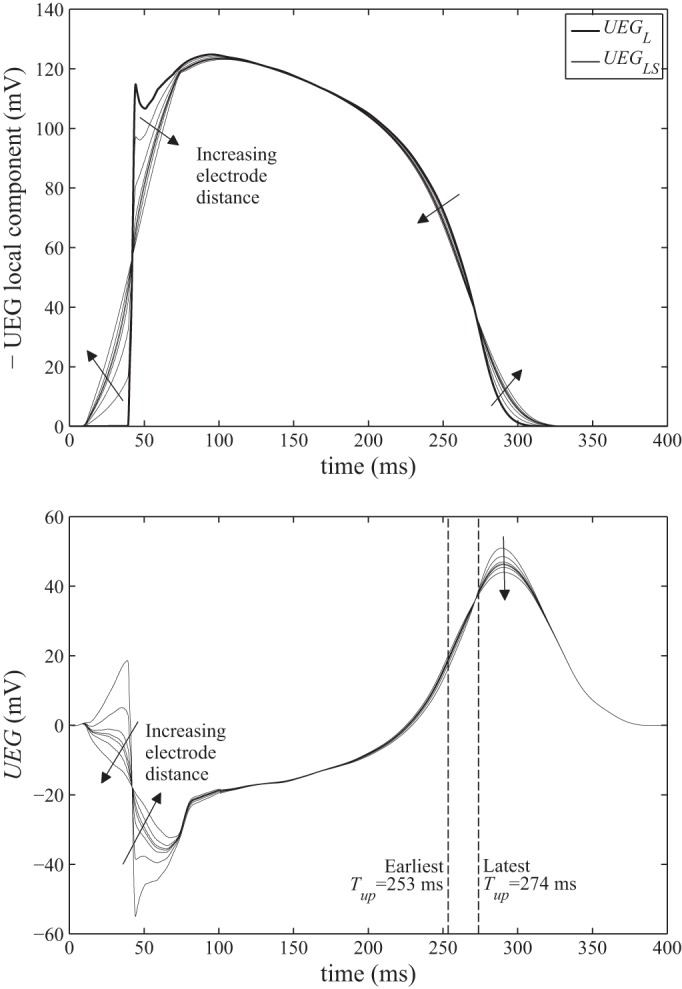
*Top*: local surface components (*UEG*_LS_) calculated using *Eq. 5* with the distance to the tissue surface, *z*, varying from 0.004 to 16 mm. For comparison, the local component used by Ref. 19 (*UEG*_L_, whose morphology is identical to that of the local membrane potential) is shown in bold. Note that the traces have been inverted to emphasize their resemblance to the familiar action potential morphology. Filled arrows indicate the sense in which the signal morphology alters as the electrode distance increases. *Bottom*: electrograms calculated from the local surface components at the *top*. Dashed vertical lines indicate the earliest and latest timings of *T*_up_, which correspond to the largest (16 mm) and smallest (0.004 mm) electrode distances, respectively.

Figure 4 shows the local surface components calculated using the method described above for the high repolarization dispersion case, along with the resulting electrograms calculated from *Eq. 5*. As the distance between the exploring electrode and the tissue surface increases, the morphologies of *UEG*_LS_ and the resulting electrogram become more “smoothed,” as predicted.

**Fig. 5.**
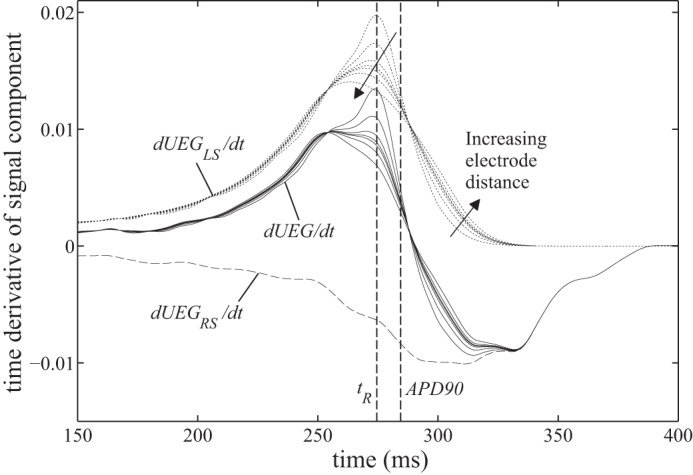
Variation in the electrogram's time derivative during repolarization (i.e., T-wave slope), as the distance from the exploring electrode to the tissue surface varies from 0.004 to 16 mm, for the high dispersion case. The electrogram's time derivative, d*UEG*/d*t*, can be calculated as the sum of the 2 components' derivatives, d*UEG*_LS_/d*t* (dotted lines) and d*UEG*_RS_/d*t* (dashed line), which are shown to clarify their separate contributions to d*UEG*/d*t*.

**Fig. 6.**
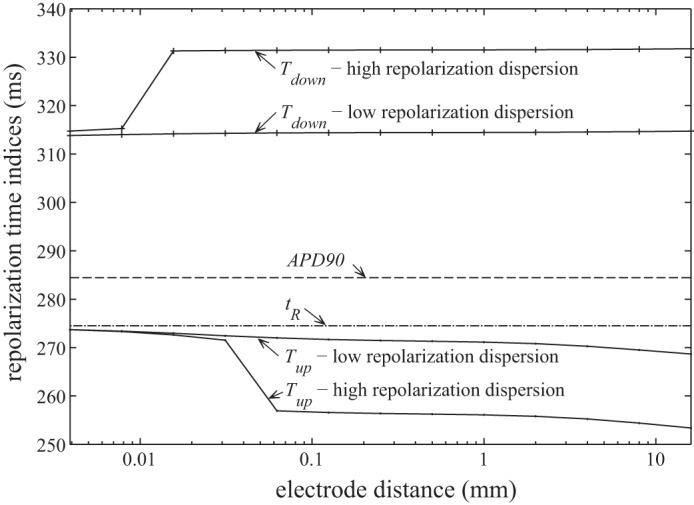
Repolarization indexes plotted against electrode distance. *t*_R_ and APD_90_ are calculated directly from the action potential morphology (*V*_m_), and are therefore independent of electrode distance.

Figure 5 shows how the slope of the T wave in *UEG* varies between these electrograms. Also shown are the slopes of the two components *UEG*_LS_ and *UEG*_RS_ to expose their separate contributions to the overall morphology (d*UEG*/d*t* = d*UEG*_RS_/d*t* − d*UEG*_LS_/d*t*). As the electrode distance increases, the peak in d*UEG*/d*t* (*T*_up_) clearly shifts from ∼274 ms to 253 ms. Similarly, the minimum slope (*T*_down_) shifts from ∼315 ms to 332 ms. These transitions are plotted against electrode distance in Figure 6, along with corresponding results from the low repolarization dispersion case. The error between *T*_up_ and the idealized measurement it is intended to reflect, *t*_R_, increases monotonically with increasing electrode distance. The same is true when comparing *T*_down_ and APD_90_, although the error between these indexes is substantial (∼30 ms) even when the electrode distance is very small, and larger errors appear as electrode distance increases. In general, the effects are reduced when the dispersion of repolarization is lower. For small electrode distances, the difference between the high and low dispersion cases is negligible, but at larger distances doubling the dispersion of repolarization is found here to increase errors by a factor greater than three.

## DISCUSSION

These results confirm the hypothesis that a positive T-wave bias emerges as a systematic consequence of increased distance between the exploring electrode and the underlying tissue. For positive T waves, the slope of the remote surface component can be expected to be increasingly negative during local repolarization, such that the maximum slope of the UEG (*T*_up_) is shifted earlier in time. Because this mechanism's emergence is indirectly dependent on T-wave polarity, we refer to it as the “polarity-dependent mechanism.”

Close inspection of Fig. 5 reveals a second, unforeseen mechanism acting in tandem with the hypothesized mechanism. Because the characteristic upward deflection in d*UEG*_LS_/d*t* is asymmetrical about its peak, the smoothing effect of increased electrode distance causes that peak to shift earlier in time. The asymmetry in d*UEG*_LS_/d*t* reflects the fact that the onset of repolarization occurs more gradually than the termination of repolarization in the action potential morphology. Virtually all myocardial action potential morphologies share this characteristic; hence, this particular mechanism of ARI shortening can be expected to occur in the majority of UEGs, albeit with variable magnitude. We refer to it as the “asymmetric smoothing mechanism.”

To recapitulate: the simulations have exposed two error mechanisms affecting recovery time estimates from UEGs. The polarity-dependent mechanism's manifestation is dependent on the relative timing of the repolarization artifacts in the local and remote surface components. The asymmetric smoothing mechanism is not directly dependent on any aspect of the remote surface component. However, it can influence the magnitude of the polarity-dependent mechanism's effects. The apparent step change in *T*_up_, observable in Fig. 6 where electrode distance is ∼0.04 mm, occurs when the asymmetric smoothing mechanism shifts *T*_up_ forward enough to coincide with a period of particularly high curvature in *UEG*_RS_. Such periods are discernible in Fig. 5 as the steepest sloping portions of d*UEG*_RS_/d*t*. The potential effect of the polarity-dependent mechanism on *T*_up_ is greatest during these periods. However, the robustness of the local surface component to this influence is variable, evidenced by the fact that this step change does not occur in the low dispersion case.

This reasoning highlights the importance of the remote surface component in these systematic errors. It can be seen that the most extreme effects of the polarity-dependent mechanism will be reduced if the recovery stage of *UEG*_RS_ contains no periods of extreme curvature. Although the focus of this article has been on manipulations of the exploring electrode, the morphology of *UEG*_RS_ is similarly dependent on the reference electrode configuration. Hence, one approach to reducing errors in *T*_up_ would be by suitable positioning of the reference electrode to ensure that *UEG*_RS_ has a smooth morphology. For example, this positioning could be chosen such that the lead field strength around the heart's surface is evenly distributed across a wide area in which repolarization times are smoothly dispersed. Further modeling studies are required to identify the most appropriate reference electrode configurations to achieve this effect.

In general, the close relationship between *T*_up_ and *t*_R_ holds only when the exploring electrode is positioned very close to the myocardium. This caveat was previously suggested by Coronel et al. (5), although the systematic nature of the associated errors has not previously been identified. The fact that increased electrode distance systematically shortens ARI in positive T-wave UEGs is presumably a prime reason why studies using highly practical, nonideal electrode configurations (1, 24) found *T*_up_ to be more problematic than highly controlled laboratory experiments (4) and computational studies (3, 19, 20) did. These more idealized studies make use of minimal electrode-tissue distances and small or infinitesimal electrode sizes. The effect of using a large electrode can be considered similar to that of using an infinitesimal virtual electrode positioned at the center of the true electrode (i.e., removed from the tissue surface, even when embedded in the myocardium). The insights from the use of *UEG*_LS_ should be borne in mind when relating the results of computational studies to practical recording arrangements.

Although the present study supports the notion that ARIs calculated from *T*_up_ are prone to a substantial systematic bias (at least in nonideal conditions), this observation should not be used to support the use of the alternative method, which employs *T*_down_ in place of *T*_up_ for positive T waves. The results presented here support previous assertions (4, 19) that *T*_down_ does not reliably track either *t*_R_ or APD_90_. In fact, it appears to be susceptible to a similar electrode-distance effect to that seen for *T*_up_, but acting in the opposite sense, with *T*_down_ sometimes occurring long after all local activity has ceased (see Fig. 5 and Fig. 6).

This study should not be used to infer any specific guidelines regarding maximum electrode size or distance for UEG recordings from which ARIs are to be calculated. The relationship between these parameters and the magnitude of the observed bias will be highly dependent on the lead field and the spatiotemporal distribution of activity. However, from the improved understanding of the mechanisms underlying the bias, one can infer that increased electrode distance will generally increase the artificial shortening of ARI in UEGs with positive T waves. It may therefore be possible to make an informed decision about the reliability of ARI measurements from a particular recording based on close inspection of the electrogram morphology and its time derivative, as shown in Fig. 5; when *T*_up_ is calculated from a UEG for which the time derivative presents a broad or fragmented peak during the T wave, the resulting ARI measurement will be more likely to have been artificially shortened. Furthermore, comparing the high and low repolarization dispersion cases in Fig. 6, it can be seen that the errors are increased when local repolarization dispersion is greater, as is likely in pathological cases.

The utility of the new expression for the electrogram's local component, *UEG*_LS_, is demonstrated in the fact that it could be used to predict the mechanisms of positive T-wave bias. Compared with the prior approach of expressing this component in terms of the action potential morphology at an infinitesimal point (2, 19), *UEG*_LS_ is better suited to assessing the influence of practical considerations such as electrode size and placement, local tissue structure, and the localized spatiotemporal distribution of activity. As indicated by the results presented here, it is essential to take such effects into account when considering how the insights from computational studies and highly controlled experiments translate into less idealized recording scenarios.

Furthermore, it should be noted that the use of *UEG*_RS_ and *UEG*_LS_ (in place of *UEG*_S_ and *UEG*_L_) is also compatible with the four component model of the UEG, derived by Colli Franzone et al. (2). Their adapted derivation incorporates the concept of the oblique dipole layer activation wavefront into *Eq. 12* and allows for heterogeneous tissue properties. As a result, two additional nonzero terms, *UEG*_A_ and *UEG*_T_, emerge from the second integral in *Eq. 12*, such that

(8) $$UEG=UEG_{\text{S}}+UEG_{\text{L}}+UEG_{\text{A}}+UEG_{\text{T}}$$

Substituting the newly developed terms gives

(9) $$UEG=UEG_{\text{RS}}+UEG_{\text{LS}}+UEG_{\text{A}}+UEG_{\text{T}}$$

Axial component:

(10) $$UEG_{\text{A}}=\int_{H}{\overset{⃑}{J}}_{a}\cdot \nabla Z\text{d}V$$

Tissue component:

(11) $$UEG_{\text{T}}=\int_{H}V_{\text{m}}\left(M\nabla \alpha \right)\cdot \nabla Z\text{d}V$$

The axial component describes the special influence of ${\overset{⃑}{J}}_{a}$, the additional current source density in the axial direction of the fibers (additional to the current source density in the direction of wavefront propagation). It plays an important role in determining the precise morphology of the UEG's activation complex. The tissue component captures the special influence of regions in which tissue properties are highly discontinuous (i.e., |∇α|≫0). For more detailed interpretation of these components, see Ref. 2.

**Fig. 7.**
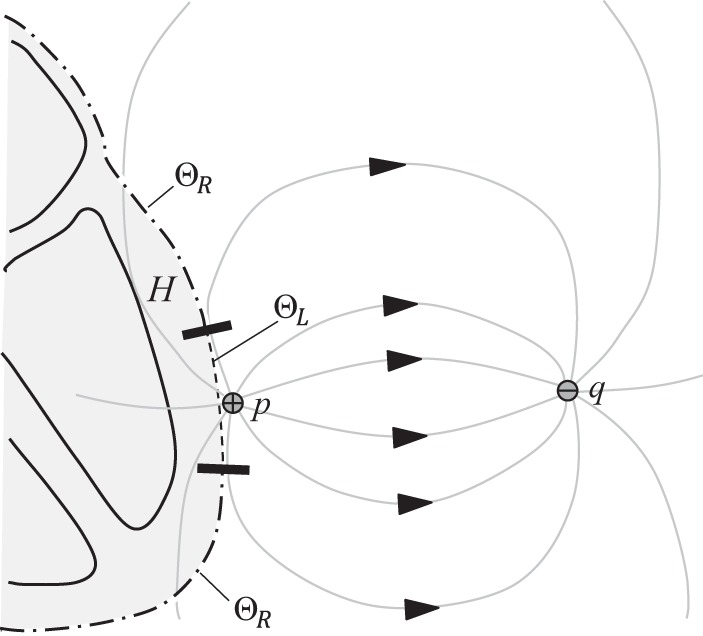
Conceptual diagram of the lead field for an epicardiac electrogram. This case differs from that in that the second boundary surface, Θ_L_, sits on the heart's outer surface, and this region is excluded from Θ_R_. Neither Θ_R_ nor Θ_L_ forms a closed surface individually, but their union does.

A notable limitation of the new expression for the local component, *UEG*_LS_, is that it lacks the simplicity of *UEG*_L_; its application requires a familiarity with the lead field concept. The lead field distribution within the heart can be strongly influenced by the varying conductive properties of the myocardium as well as surrounding tissues; the lungs are notable for having a high electrical impedance that varies with breathing. For this reason, further modeling work would be useful in providing a greater familiarity with the typical behavior of the lead field within and around the heart. For clarity of presentation, the conceptual diagrams in Figs. 2 and 7 do not take such influences into account.

### Conclusions

Building on analytic expressions developed by previous authors, this study presents a new expression for the electrogram's local component, *UEG*_LS_. Compared with prior expressions, this form is particularly suited to considering the impact of clinical experimental limitations such as electrode size and position on electrogram morphology. The expression is used to predict that a bias arises when ARI is calculated from electrograms with positive T waves. Evidence of this bias exists in the literature and has provoked debate, but its genesis has not previously been explained. The explanation given in this article reconciles the apparent discrepancies between previous works (5, 25) by confirming that they largely stem from differences in recording setup.

The new expression for the UEG's local component provides a means of understanding the influences of practical considerations on the morphology of these recordings and the reliability of any measures derived from them. The utility of this approach is demonstrated in this article. We anticipate that this formulation can be further employed to inform the development of more reliable surrogate measures of localized electrophysiological behavior based on electrogram recordings and to facilitate wider use of analytic representations of UEG morphology in the interpretation of computational results and practical observations.

## GRANTS

This work was supported by the Engineering and Physical Sciences Research Council through a doctoral training account allocated by University College London Mechanical Engineering.

## DISCLOSURES

No conflicts of interest, financial or otherwise, are declared by the author(s).

## AUTHOR CONTRIBUTIONS

Author contributions: D.G.W., B.H., and P.T. conception and design of research; D.G.W. performed experiments; D.G.W. analyzed data; D.G.W., B.H., and P.T. interpreted results of experiments; D.G.W. prepared figures; D.G.W. drafted manuscript; D.G.W., B.H., and P.T. edited and revised manuscript; D.G.W., B.H., and P.T. approved final version of manuscript.

## Appendix

For the task of interpreting UEG morphology, the most useful theoretical descriptions stem from the bidomain model, which is the most realistic model of cardiac electrophysiology for which whole heart simulations are currently computationally tractable (7, 9, 14, 16, 22). This model treats the intracellular and extracellular domains as continuous media that both occupy the whole of the myocardium, with their effective properties scaled by a factor to account for the actual geometric relationship between the two domains. The two domains interact by the exchange of membrane current.

From the bidomain model, Geselowitz (8) derived a succinct expression for the UEG. *Equation 12* presents this expression in the more general format used by Colli Franzone et al. (2) to account for heterogeneity in the anisotropic conductive properties of the myocardium.

(12) $$UEG=\int_{\Theta }\alpha V_{\text{m}}M\nabla Z\cdot \text{d}\overset{⃑}{S}+\int_{H}\alpha V_{\text{m}}\nabla \cdot \left(M\nabla Z\right)\text{d}V$$

On the right-hand side, the first term is an integral around the heart's outer surface, Θ. The second term is an integral throughout *H*, the volume contained by Θ. d*V* is an infinitesimal volume element of *H*.

Geselowitz (8) noted that the integrand of the second term in *Eq. 12* is equal to 0 throughout *H*, except for a singularity at the site of any electrode. As a result, for a typical unipolar electrode configuration where the “reference” electrode is outside Θ but the “exploring” electrode is at some point *p* inside Θ, *Eq. 12* reduces to

(13) $$UEG=\int_{\Theta }\alpha V_{\text{m}}M\nabla Z\cdot \text{d}\overset{⃑}{S}-\alpha V_{\text{m}}\left(p\right)$$

The simplicity of this expression is attractive for the purposes of analytic interpretations of UEG morphology, because it allows the signal to be considered in terms of a surface component, *UEG*_S_, and a “local” component, *UEG*_L_:

(14) $$UEG_{\text{S}}=\int_{\Theta }\alpha V_{\text{m}}M\nabla Z\cdot \text{d}\overset{⃑}{S}$$

(15) $$UEG_{\text{L}}=-\alpha V_{\text{m}}\left(p\right)$$

The local component is expressed simply as a scaled, inverted version of the local action potential morphology. The surface component is effectively a weighted average of the action potential morphologies around the heart's surface, and accounts for the influence of “remote” activity. It should be noted here that Potse et al. (19) refer to this as the “remote component.” We use the term “surface component” because, in the more detailed formulation used by Colli Franzone et al. (2), *UEG*_S_ also features but is not the only term that can encapsulate remote influences.

Potse et al. (19) demonstrated through computer simulations with a realistic whole heart model that in healthy hearts the morphology of *UEG*_S_ does not vary substantially for different electrode configurations; it resembles a smoothed action potential, as depicted in Fig. 1. These authors also demonstrated that the polarity of the UEG's T wave (as well as the activation wave) is dependent on the relative timing of features in the local and surface components, with positive T waves arising from sites where local repolarization occurs relatively early (19), as is the case in Fig. 1.

These insights are extremely useful in enabling intuitive predictions of UEG morphology based on the basic spatiotemporal distribution of activity. However, the simplicity of *UEG*_L_ as expressed in *Eq. 14* limits its utility; clearly, it provides no insight as to how electrogram morphology varies according to the size or position of the exploring electrode relative to the local tissue. Furthermore, when the exploring electrode is not embedded in the myocardium, for example, when it sits in the intracavitary space or in loose contact with the myocardial surface, *V*_m_(*p*) cannot be sensibly defined as there are no myocytes at the electrode position. Yet, experience and intuition indicate that an electrode positioned just outside a region of active myocardium is still sensitive to the activity of that region. To facilitate an intuitive expression for the UEG's local component in such scenarios, we propose the following adapted derivation. The derivation is presented with reference to the case in which the exploring electrode is located in the endocardial cavity. Subsequently, we explain how the model can be adapted to mid-myocardial and epicardial electrode positions.

### Endocardial Electrograms

If Θ, the bounding surface of *H*, is the union of two nonoverlapping surfaces Θ_R_ and Θ_L_, then the full surface component (see *Eq. 14*) can be rewritten as

(16) $$UEG_{\text{S}}=\int_{\Theta_{\text{R}}\cup \Theta_{\text{L}}}\alpha V_{\text{m}}M\nabla Z\cdot \text{d}\overset{⃑}{S}=\int_{\Theta_{\text{R}}}\alpha V_{\text{m}}M\nabla Z\cdot \text{d}\overset{⃑}{S}+\int_{\Theta_{\text{L}}}\alpha V_{\text{m}}M\nabla Z\cdot \text{d}\overset{⃑}{S}$$

For the endocardial case, consider Θ_R_ to be the outer surface of the heart (previously Θ). Let Θ_L_ be defined as the endocardial wall of the chamber containing the exploring electrode, acting as an interior boundary surface of *H* such that *p* is excluded from *H* (see Fig. 2). With both electrodes outside of *H*, the local component does not emerge from the derivation of *UEG*; there is no singularity in the second integrand of *Eq. 12*. However, the surface component *UEG*_S_ now consists of two subcomponents, *UEG*_RS_ and *UEG*_LS_, as defined in *Eqs. 2* and *3*.

(17) $$UEG=UEG_{\text{S}}=UEG_{\text{RS}}+UEG_{\text{LS}}$$

Note that in this case *UEG*_RS_ is identical to *UEG*_S_ as expressed in *Eq. 14*. *UEG*_LS_ is referred to as the “local surface component”; it effectively replaces *UEG*_L_ in the definition of *UEG*, with the advantage that it allows consideration of the local tissue properties, the local spatiotemporal distribution of activity, and the local characteristics of the lead field, which is partly determined by the position of the electrode relative to the tissue.

As long as Θ_R_ and Θ_L_ are chosen such that all current sources, that is, all active cardiac myocytes, are still contained in *H*, then no additional terms arise from this manipulation.

### Mid-Myocardial Electrograms

*UEG*_LS_ can also be used to analyze electrodes embedded in the myocardium if Θ_L_ is taken as the boundary surface between the electrode and the tissue. This approach is useful in cases where the approximation of the electrode as an infinitesimal point is deemed too simplistic, for example, when assessing the effects of an electrode's size or shape on the measurement.

### Epicardial Electrograms

When the exploring electrode is positioned outside the heart, it is not appropriate to use an interior boundary surface to calculate the electrogram's local component, as it will be remote from the exploring electrode. An appropriate alternative is to choose the second boundary surface Θ_L_ as a portion of the heart's outer boundary, in the vicinity of the exploring electrode. Θ_L_ can be more precisely defined as the continuous region in which all lead field lines cross the heart's outer surface in the inward direction (Fig. 7). This definition ensures that, in regions where excitation progresses smoothly, *UEG*_LS_ retains the expected morphology of a smoothed, inverted action potential. Θ_R_ should then be redefined as the heart's outer boundary, excluding Θ_L_ so that *Eq. 16* still holds.

## Glossary

APD: Action potential duration
APD_90_: Repolarization time, defined as the time at which the action potential morphology recovers 90% of the difference between the polarized and depolarized (plateau) potentials
ARI: Activation-recovery interval, a surrogate measure of APD, taken from the UEG
d*V*/d*t*_min_: Activation time, measured from the *UEG* as the steepest downstroke of the activation complex
*H*: The volume contained within Θ (or within Θ_R_ ∪ Θ_L_)
${\overset{⃑}{J}}_{a}$: The component of the dipole current source density in the axial direction of the myocardial fibers
*M*: Conductivity tensor
MAP_90_: Repolarization time, measured from the monophasic action potential signal using the same definition as for APD_90_.
*T*_down_: Recovery time, measured from the *UEG* as the steepest downstroke of the T wave (alternative method)
*T*_up_: Recovery time, measured from the *UEG* as the steepest upstroke of the T wave (Wyatt method)
*t*_R_: Repolarization time, measured from the action potential morphology as the steepest downstroke in phase 3
UEG: Unipolar electrogram
*UEG*: The unipolar electrogram's voltage signal (italicized when used in mathematical descriptions)
*UEG*_L_: *UEG*'s local component
*UEG*_S_: *UEG*'s (remote) surface component, used with *UEG*_L_
*UEG*_RS_: *UEG*'s remote surface component, used with *UEG*_LS_
*UEG*_LS_: *UEG*'s local surface component, which replaces *UEG*_L_ in the new formulation
*UEG*_A_: *UEG*'s axial component
*UEG*_T_: *UEG*'s tissue component
*V*_m_: Transmembrane potential, a function of time and position within the myocardium
∇Z: Lead field; its value at each point depends on the electrode positions and the distribution of impedance properties throughout the body
α: Tissue constant
Θ: The outer surface of the heart
Θ_R_: The outer surface of the heart (excluding Θ_L_)
Θ_L_: The local myocardial surface, chosen according to the exploring electrode placement
σ: The bulk conductivity (σ_e_ + σ_i_) of the myocardium (assumed to be isotropic)
σ_t,i_: Effective conductivity of the intracellular (i) domain in the transverse (t) direction
σ_t,e_: Effective conductivity of the extracellular (e) domain in the transverse (t) direction
